# Role of ATF3 triggering M2 macrophage polarization to protect against the inflammatory injury of sepsis through ILF3/NEAT1 axis

**DOI:** 10.1186/s10020-023-00711-9

**Published:** 2024-02-23

**Authors:** Wei Wang, Rongli Xu, Ping He, Yuqing Xiong, Haomiao Zhao, Xuewei Fu, Jie Lin, Lijiao Ye

**Affiliations:** 1 Geriatric Medicine Department, The Fifth Affiliated Hospital of Southern Medical University, No. 566, Congcheng Avenue, Conghua District, Guangzhou, 510920 Guangdong Province People’s Republic of China; 2 Department of Cardiology, Hainan General Hospital, Hainan Affiliated Hospital of Hainan Medical University, Haikou, 570311 Hainan Province People’s Republic of China; 3 Department of Emergency, Hainan General Hospital, Hainan Affiliated Hospital of Hainan Medical University, Haikou, 570311 Hainan Province People’s Republic of China

**Keywords:** ATF3, ILF3, NEAT1, Sepsis, Macrophage polarization

## Abstract

**Background:**

Sepsis is a systemic inflammatory response which is frequently associated with acute lung injury (ALI). Activating transcription factor 3 (ATF3) promotes M2 polarization, however, the biological effects of ATF3 on macrophage polarization in sepsis remain undefined.

**Methods:**

LPS-stimulated macrophages and a mouse model of cecal ligation and puncture (CLP)-induced sepsis were generated as in vitro and in vivo models, respectively. qRT-PCR and western blot were used to detect the expression of ATF3, ILF3, NEAT1 and other markers. The phenotypes of macrophages were monitored by flow cytometry, and cytokine secretion was measured by ELISA assay. The association between ILF3 and NEAT1 was validated by RIP and RNA pull-down assays. RNA stability assay was employed to assess NEAT1 stability. Bioinformatic analysis, luciferase reporter and ChIP assays were used to study the interaction between ATF3 and ILF3 promoter. Histological changes of lung tissues were assessed by H&E and IHC analysis. Apoptosis in lungs was monitored by TUNEL assay.

**Results:**

ATF3 was downregulated, but ILF3 and NEAT1 were upregulated in PBMCs of septic patients, as well as in LPS-stimulated RAW264.7 cells. Overexpression of ATF3 or silencing of ILF3 promoted M2 polarization of RAW264.7 cells via regulating NEAT1. Mechanistically, ILF3 was required for the stabilization of NEAT1 through direct interaction, and ATF3 was a transcriptional repressor of ILF3. ATF3 facilitated M2 polarization in LPS-stimulated macrophages and CLP-induced septic lung injury via ILF3/NEAT1 axis.

**Conclusion:**

ATF3 triggers M2 macrophage polarization to protect against the inflammatory injury of sepsis through ILF3/NEAT1 axis.

## Introduction

Sepsis, a systemic inflammatory response, is a result of infection and frequently associated with multiple organ failure (Cecconi et al. 2018). Acute lung injury (ALI) or acute respiratory distress syndrome (ARDS) are common lethal complications of sepsis with substantial morbidity and mortality (Varisco 2011). Currently, initial infection control and organ support are therapeutic approaches for sepsis (Lelubre and Vincent 2018). Unfortunately, severe sepsis is still associated with high mortality rate and rehospitalization due to lack of effective clinical protocol (Farrah et al. 2021). Unraveling the molecular mechanism underlying sepsis-induced lung injury will provide in-depth understanding of sepsis pathogenesis and provide novel insights into the clinical practice.

Macrophages play pivotal roles in eliminating pathogens, tissue homoeostasis and anti-inflammation (Liu et al. 2014). Continued activation of macrophages is implicated in accelerated septic response (Stearns-Kurosawa et al. 2011). Activated macrophages are classified into pro-inflammatory M1 macrophages and anti-inflammatory M2 macrophages (Sica and Mantovani 2012), and macrophages interconvert their phenotypes in response to different stimuli which termed macrophage polarization (Murray 2017). It is well-established that macrophage polarization is associated with diverse inflammatory diseases, including sepsis (Liu et al. 2014). A number of studies have illustrated that different therapeutic agents ameliorate sepsis and its complications via modulating macrophage polarization (Feng et al. 2014; Li et al. 2021; Zhang et al. 2021). There is a surge of interest to investigate the regulatory mechanism of macrophage polarization for targeted therapy of sepsis.

Activating transcription factor 3 (ATF3) belongs to the ATF/cAMP response element-binding (CREB) family and functions as a stress-induced transcription factor in a variety of physiological and pathological processes (Ku and Cheng 2020). RNA sequencing has revealed the differential expression pattern of ATF3 in lipopolysaccharide (LPS)-induced ALI, and it is also identified as a potential biomarker for ALI by PPI network analysis (Luo et al. 2021). Previous study has demonstrated that ROS-induced ATF3 potentiates susceptibility to secondary infection in sepsis (Hoetzenecker et al. 2011). It suppresses LPS-induced inflammation via inhibiting HMGB1 (Lai et al. 2013). Intriguingly, ATF3 promotes macrophage migration and M2 polarization by inducing TNC via Wnt/β-catenin pathway (Sha et al. 2017). However, the biological effects of ATF3 on macrophage polarization in sepsis remain largely unknown. Bioinformatic analysis predicted the potential binding sites between ATF3 and the interleukin enhancer binding factor 3 (ILF3) promoter, and our preliminary data revealed the putative interaction between ILF3 and long non-coding RNA (lncRNA) NEAT1 in RAW264.7 cells. In addition, a recent study has demonstrated that NEAT1 promotes M2 polarization via miR-125a-5p/TRAF6/TAK1 axis, thus modulating LPS-induced inflammation (Wang and Guo 2020), supporting the role of NEAT1 in macrophage polarization during sepsis-inflammatory responses. These findings raise the possibility that ATF3/ILF3/NEAT1 axis might play a critical role in sepsis by modulating macrophage polarization.

In this study, we demonstrated that ATF3 was downregulated, but ILF3 and NEAT1 were upregulated in PBMCs derived from septic patients, as well as in LPS-stimulated macrophages. Overexpression of ATF3 or silencing of ILF3 promoted M2 polarization in LPS-treated RAW264.7 cells. Mechanistic study suggested that ILF3 was required for the stabilization of NEAT1 through direct interaction, and ATF3 was a transcriptional repressor of ILF3. In vitro functional experiments and in vivo studies in cecal ligation and puncture (CLP)-induced sepsis model revealed that ATF3 facilitated M2 polarization in sepsis via ILF3/NEAT1 axis.

## Materials and methods

### Clinical specimens

This study was approved by the Fifth Affiliated Hospital of Southern Medical University. Whole peripheral blood was collected from patients with sepsis (n = 40) or healthy volunteers (n = 40) from the Fifth Affiliated Hospital of Southern Medical University hospital. Written consents from all recruiters were obtained.

### Peripheral blood mononuclear cells (PBMCs) isolation

PBMCs isolation was conducted by Ficoll centrifugation as described (Rahmel et al. 2020). Briefly, cells were centrifuged in Ficoll-Paque media (Cytiva, Marlborough, MA, USA). PBMCs were collected and directly subjected to subsequent analysis.

### Cell culture, treatment and transfection

Mouse RAW264.7 macrophages were from ATCC (Manassas, VA, USA), and cultured in DMEM containing 10% FBS (Gibco, Great Island, NY, USA). Cells were maintained at 37 °C with 5% CO_2_ in air. For the in vitro cell model, RAW264.7 cells were treated with 10 μg/mL lipopolysaccharide (LPS, Sigma-Aldrich, St. Louis, MO, USA) for 24 h. OE-ATF3, OE-ILF3, pcDNA-NEAT1, sh-NC, sh-ILF3 were obtained from GenePharma (Shanghai, China). RAW264.7 cells were transfected with overexpression construct and/or shRNA using Lipofectamine 3000 (Invitrogen, Carlsbad, CA, USA), followed by the stimulation of LPS at 48 h post-transfection.

### qRT-PCR

Total RNA was extracted using Trizol (Invitrogen). cDNA was synthesized using HiScript 1st Strand cDNA Synthesis Kit (Vazyme, Nanjing, China). The relative expression of target gene was detected using iQ SYBR Green Supermix (Bio-Rad, Hercules, CA, USA) and determined using 2 ^–ΔΔCT^ method. For RNA stability assay, cells were treated with actinomycin D (2 μg/ml, Sigma-Aldrich) for 0, 1, 2, 3 and 4 h. The expression of NEAT1 was detected by qRT-PCR. The primers used in qRT-PCR was listed in Table 1.

**Table 1:** The primers used in qRT-PCR

Primer	Sequence 5’-3’
ATF3 sense	GTGCCGAAACAAGAAGAAGG
ATF3 anti-sense	TCTGAGCCTTCAGTTCAGCA
ILF3 sense	GTGTCCAATCACCAGTCCTG
ILF3 anti-sense	GCTGAAGAAGTGGGAGTGTAGC
NEAT1 sense	GGGGCCACATTAATCACAAC
NEAT1 anti-sense	CAGGGTGTCCTCCACCTTTA
TNF-α sense	CCGATGGGTTGTACCTTGTC
TNF-α anti-sense	TGGAAGACTCCTCCCAGGTA
IL-1β sense	AGGAGCTGTCATTAGGGACAT
IL-1β anti-sense	AAGGTCCACGGGAAAGACAC
IL-6 sense	TGCAAGAGACTTCCATCCAG
IL-6 anti-sense	TCCACGATTTCCCAGAGAAC
iNOS sense	CCAAGCCCTCACCTACTTCC
iNOS anti-sense	GGCAGTGTAACTCTTCTGCAT
IL-4 sense	CCTCACAGCAACGAAGAACA
IL-4 anti-sense	TGGACTCATTCATGGTGCAG
IL-10 sense	GGTTGCCAAGCCTTATCGGA
IL-10 anti-sense	TTCAGCTTCTCACCCAGGGA
Arg-1 sense	CTCCAAGCCAAAGTCCTTAGAG
Arg-1 anti-sense	AGGAGCTGTCATTAGGGACAT
ATF3 (for ChIP) sense	TGGCAACACGGAGTAAACGAC
ATF3 (for ChIP) anti-sense	AGAGAAGAGAGCTGTGCAGTGC
GAPDH sense	CCAGGTGGTCTCCTCTGA
GAPDH anti-sense	GCTGTAGCCAAATCGTTGT

### Western blot

Protein lysates were prepared using RIPA buffer (Pierce, Rockford, IL, USA), and separated by gel electrophoresis. Proteins were then transferred onto PVDF membrane (Pierce). After blocking, the blots were incubated with appropriate primary and secondary antibodies. The protein bands were detected suing ECL reagents (Pierce). The intensities of bands were quantified using Image J software (NIH) and normalized with GAPDH. Primary antibodies used in this study: anti-ATF3 (1:1000, ab207434, Abcam, Cambridge, UK), anti-ILF3 (1:2000, ab131004, Abcam), anti-iNOS (1:1000, ab178945, Abcam), anti-Arg-1 (1:5000, PA529645, Invitrogen) and anti-GAPDH (1:2000, ab8245, Abcam) antibody.

### Flow cytometry

RAW264.7 cells were stained with anti-F4/80, iNOS and CD206 antibodies (Invitrogen). Briefly, cells were stained with cell surface marker prior to fixation and permeabilization with eBioscience Intracellular Fixation & Permeabilization Buffer (88–8824-00, Invitrogen). Cells were then stained with intracellular marker and analyzed using BD flow cytometer (BD Biosciences, San Jose, CA, USA).

### RNA immunoprecipitation (RIP) assay

RIP was conducted using Magna RIP Kit (Millipore, Billerica, MA, USA). In brief, cells were lysed with RIP lysis buffer. Anti-ILF3 (2 μg, ab131004, Abcam) or normal rabbit IgG-conjugated beads were then incubated with cell lysates. qRT-PCR was employed to detect immunoprecipitated NEAT1. LincIN served as a positive control.

### RNA pull-down assay

RNA pull-down assay was carried out using Pierce RNA Pull-Down Kit (Pierce). The wide-type or mutated NEAT1 probe (NEAT1-WT or NEAT1-MUT) was labeled with desthiobiotin and conjugated with streptavidin beads. The beads were then incubated with cell lysates, and the enriched proteins were eluted and analyzed by western blot. Anti-sense NEAT1 probe acted as a negative control.

### Nascent RNA capture assay

Nascent RNA was prepared using Click-iT Nascent RNA Capture Kit (Invitrogen). In brief, EU labeled RNA was conjugated to biotin azide via a click reaction. The biotinylated RNA was then enriched using Dynabeads MyOne streptavidin beads, and subjected to reverse transcription and qRT-PCR analysis as above mentioned.

### Dual luciferase reporter assay

The promoter region p(-2071/-2078) of ILF3 was cloned into pGL-3 vector (Promega, Madison, WI, USA). The luciferase construct and ATF3 overexpression plasmid were co-transfected into RAW264.7 cells. Luciferase activity was measured using Dual Luciferase Assay System (Promega) at 48 h post-transfection.

### Chromatin immunoprecipitation (ChIP) assay

ChIP assay was conducted using Pierce Agarose ChIP Kit (Pierce). Briefly, crosslinked RAW264.7 cells were lysed and digested by MNase. The chromatin fractions were then incubated with anti-ATF3 (2 μg, ab207434, Abcam) or normal rabbit IgG conjugated beads. DNA were further purified and analyzed by PCR.

### Animal study and sampling

Animal study was approved by the Fifth Affiliated Hospital of Southern Medical University. Male C57BL/6 mice (25–30 g, n = 6 per group) were obtained from the Fifth Affiliated Hospital of Southern Medical University. Mice were randomly divided into five groups: Sham, CLP, CLP + OE-NC, CLP + OE-ATF3 and CLP + OE-ATF3 + ILF3. To establish the cecal ligation and puncture (CLP)-induced sepsis model, the mice were anesthetized, and the disinfected abdominal region was incised. The cecum was isolated, and the distal thirds of the cecum were tightly ligated and punctured with a 30-gauge needle. The cecum was repositioned and the abdomen was closed subsequently. For the Sham group, mice underwent the same surgery without ligation and puncture. At 1 h post-surgery, transfected RAW264.7 cells were injected into the tail vein. The survival analysis was conducted using Kaplan–Meier method. At 12 h post-CLP surgery, blood sample was collected and analyzed on a Blood Cell Analyzer (Beckman Coulter, Brea, CA, USA). Total protein concentration was determined using Tripure Isolation Reagent (Roche). Mice were sacrificed at 24 h after CLP surgery, and the lungs and bronchoalveolar lavage fluid (BALF) were collected for subsequent analysis. To assess lung edema, lung wet to dry (W/D) ratio was calculated as previous described (Luo et al. 2020).

### ELISA assay

The levels of TNF-α (88-7324-22, Invitrogen), IL-1β (88-7013-22, Invitrogen), IL-6 (88-7064-22, Invitrogen) and IL-4 (88-7044-22, Invitrogen) in BALF and lung tissues were detected using commercial kits. ELISA assay was conducted according to the manufacturer’s instructions, and A450 was measured using a microplate reader (Thermo Fisher Scientific).

### Histological analysis

The lungs were fixed and embedded in paraffin. After the deparaffinization and rehydration. The slides were then stained with hematoxylin & eosin (H&E). For IHC, the sections were subjected to antigen retrieval and blocking. This was followed by the incubation with appropriate primary and secondary antibodies. Signal was visualized using the DAB substrate (Pierce). Primary antibody used in IHC: anti-CD163 (1:200, ab182422, Abcam) antibody.

### TUNEL assay

DNA fragmentation was monitored by TUNEL assay. In brief, the sections were fixed and permeabilized. The slides were then incubated with TUNEL reaction buffer (Roche, Indianapolis, IN, USA) at 37 °C for 2 h. Signals were detected using DAB substrate (Pierce).

### Statistical analysis

All the experiments were repeated at least three times. Statistical analysis was conducted using GraphPad Prism 8.1.2 (GraphPad, La Jolla, CA, USA). Student’s *t*-test or one-way ANOVA was used to assess the differences for two-groups or multiple-groups comparison, respectively. *P* < 0.05 were considered significant.

## Results

### Expression patterns of ATF3, ILF3 and NEAT1 in sepsis

**Fig. 1:**
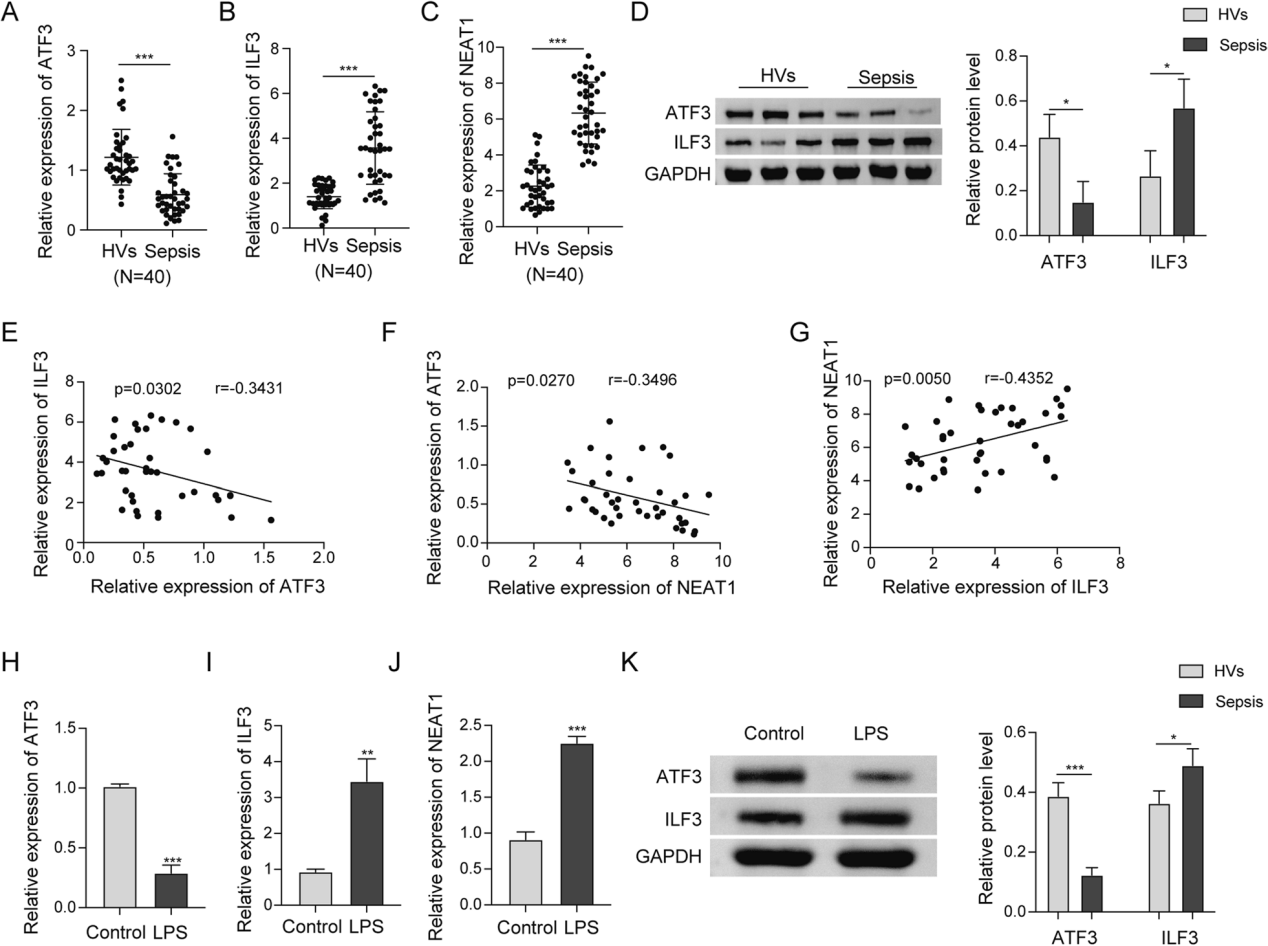
Expression patterns of ATF3, ILF3 and NEAT1 in sepsis. **a**–**c** The expression of ATF3, ILF3 and NEAT1 in the PBMCs derived from healthy volunteers (HVs) and septic patients were detected by qRT-PCR. **d** The protein levels of ATF3 and ILF3 in the PBMCs derived from HVs and septic patients were detected by western blot. **e**–**g** The correlations among ATF3, ILF3 and NEAT1 in PBMCs from septic patients were analyzed by Spearman correlation analysis. **h**–**j** The expression of ATF3, ILF3 and NEAT1 in LPS-treated RAW264.7 cells were detected by qRT-PCR. **k** The protein levels of ATF3 and ILF3 in LPS-treated RAW264.7 cells were detected by western blot. *, *P* < 0.05, **, *P* < 0.01, ***, *P* < 0.001

To investigate the biological roles of ATF3/ILF3/NEAT1 axis, we first examined the expression of these molecules in sepsis. As presented in Fig. 1a–c, qRT-PCR showed that ATF3 was downregulated, but ILF3 and NEAT1 were upregulated in the PBMCs of septic patients, compared with that of healthy donors. Western blot revealed the decreased expression of ATF3, alone with the increased expression of ILF3 in the PBMCs derived from patients with sepsis (Fig. 1d). Spearman correlation analysis showed that there was a negative correlation between ILF3 and ATF3, as well as between ATF3 and NEAT1 in PBMCs from septic patients (Fig. 1e, f). By contrast, a positive correlation between NEAT1 and ILF3 was observed in PBMCs from patients with sepsis (Fig. 1g). In consistent with the clinical data, ATF3 was decreased, whereas ILF3 and NEAT1 were increased in LPS-stimulated RAW264.7 cells (Fig. 1h–k). The reduction of ATF3 and induction of ILF3 in LPS-treated RAW264.7 cells were further confirmed by western blot (Fig. 1k). These findings suggest that ATF3 was reduced, while ILF3 and NEAT1 were elevated in sepsis.

### Reinforced ATF3 promotes M2 polarization of RAW264.7 cells upon LPS treatment

**Fig. 2:**
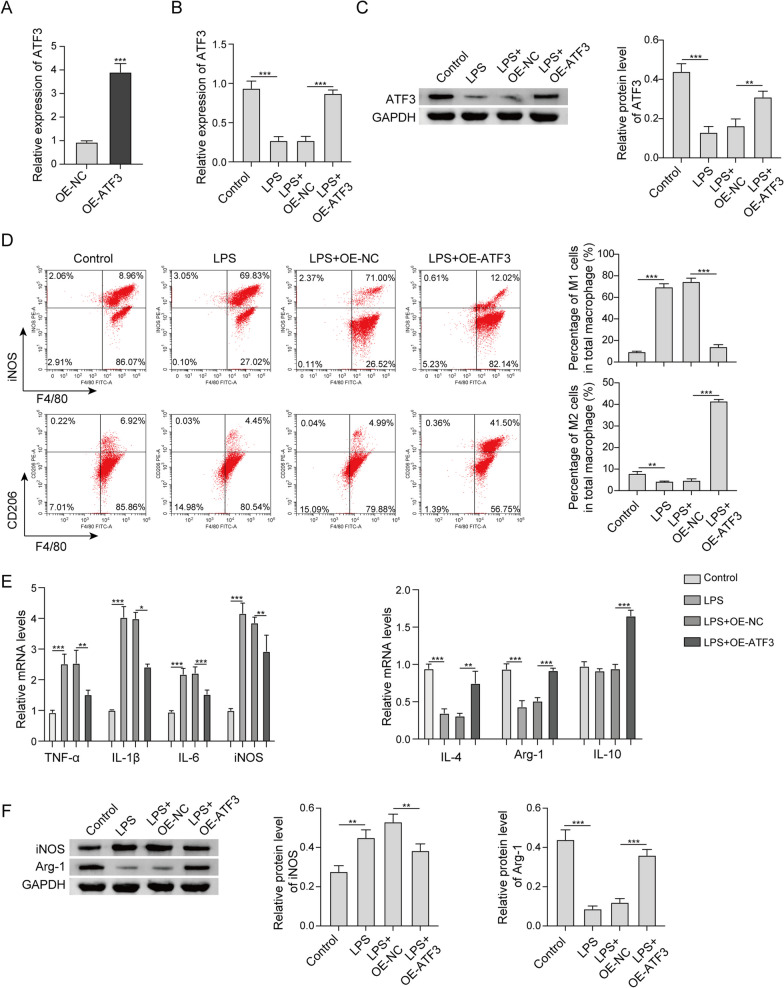
Reinforced ATF3 promotes M2 polarization of RAW264.7 cells upon LPS treatment. **a**, **b** The mRNA level of ATF3 in RAW264.7 cells was detected by qRT-PCR. **c** The protein level of ATF3 in RAW264.7 cells was detected by western blot. **d** The percentages of M1/M2 macrophages were analyzed by flow cytometry. **e** The mRNA levels of M1 and M2 markers in RAW264.7 cells were detected by qRT-PCR. **f** The protein levels of iNOS and Arg-1 in RAW264.7 cells were detected by western blot. *, *P* < 0.05, **, *P* < 0.01, ***, *P* < 0.001

Overexpression studies were next carried out to investigate the effects of ATF3 on M2 polarization. As expected, transfection of ATF3 overexpression plasmid successfully induced ATF3 expression in RAW264.7 cells (Fig. 2a). LPS remarkably reduced ATF3 expression, while ATF3 overexpression led to a rebound of ATF3 at both mRNA and protein levels (Fig. 2b, c). As shown in Fig. 2d, LPS increased the population of F4/80^+^iNOS^+^ M1 macrophages, accompanied with the decreased proportion of F4/80^+^CD206^+^ M2 macrophages. Overexpression of ATF3 dramatically reversed the effect of LPS on M2 polarization (Fig. 2d). In line with these findings, the classical M1 markers TNF-α, IL-1β, IL-6 and iNOS were increased by LPS, and these effects were abrogated by ATF3 overexpression. The LPS-mediated reduction of M2 markers IL-4 and Arg-1 were also counteracted by ATF3 overexpression (Fig. 2e), whereas IL-10 level was not changed within IPS exposure. In addition, the changes of iNOS and Arg-1 were also validated by western blot (Fig. 2f). Collectively, these data indicate that ATF3 facilitated M2 polarization of macrophages upon LPS treatment.

### NEAT1 is a downstream effector to be involved in ATF3-mediated M2 polarization

**Fig. 3:**
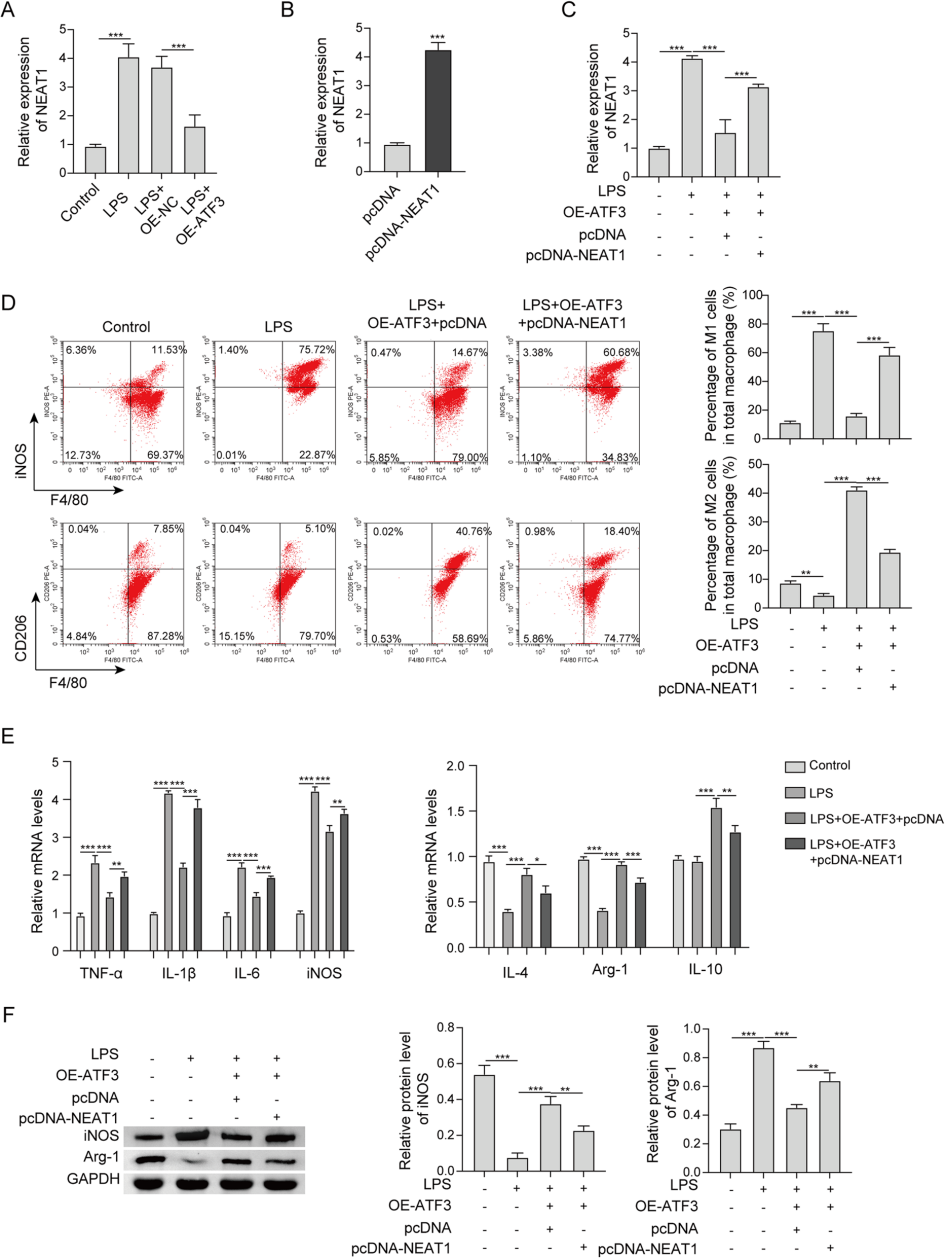
NEAT1 is a downstream effector to be involved in ATF3-mediated M2 polarization. **a**–**c** The level of NEAT1 in RAW264.7 cells was detected by qRT-PCR. **d** The percentages of M1/M2 macrophages were analyzed by flow cytometry. **e** The mRNA levels of M1 and M2 markers in RAW264.7 cells were detected by qRT-PCR. **f** The protein levels of iNOS and Arg-1 in RAW264.7 cells were detected by western blot. *, *P* < 0.05, **, *P* < 0.01, ***, *P* < 0.001

Intriguingly, qRT-PCR showed that LPS-induced NEAT1 was attenuated by ATF3 overexpression (Fig. 3a). We next sought to test whether NEAT1 functioned as a downstream effector in ATF3-mediated M2 polarization. Transfection of NEAT1 overexpression construct markedly increased NEAT1 level in RAW264.7 cells (Fig. 3b), and NEAT1 overexpression counteracted ATF3-suppressed NEAT1 expression (Fig. 3c). Consistently, LPS-increased M1 macrophages were reduced in LPS + OE-ATF3 + pcDNA group, whereas this effect of ATF3 was abolished by NEAT1 overexpression. An opposite trend of M2 macrophages was also observed by flow cytometry (Fig. 3d). Similarly, ATF3-mediated changes of M1 and M2 markers were reversed, at least in part, by NEAT1 overexpression in the presence of LPS (Fig. 3e, f). These data suggest that ATF3 mediated M2 polarization via suppressing NEAT1 expression in LPS-stimulated macrophages.

### Depletion of ILF3 promotes M2 polarization of RAW264.7 cells upon LPS treatment

**Fig. 4:**
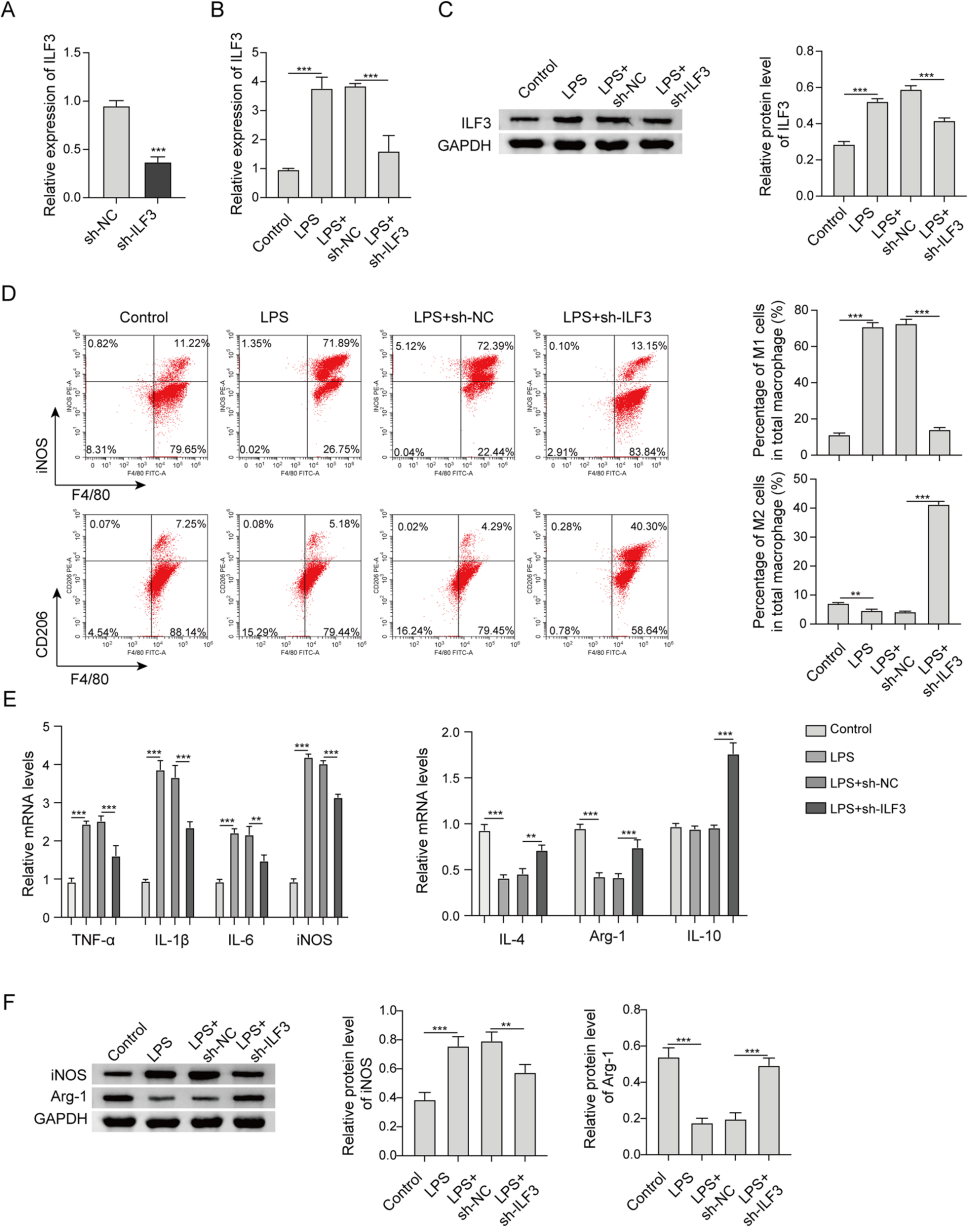
Depletion of ILF3 promotes M2 polarization of RAW264.7 cells upon LPS treatment. **a**, **b** The mRNA level of ILF3 in RAW264.7 cells was detected by qRT-PCR. **c** The protein level of ILF3 in RAW264.7 cells was detected by western blot. **d** The percentages of M1/M2 macrophages were analyzed by flow cytometry. **e** The mRNA levels of M1 and M2 markers in RAW264.7 cells were detected by qRT-PCR. **f** The protein levels of iNOS and Arg-1 in RAW264.7 cells were detected by western blot. **, *P* < 0.01, ***, *P* < 0.001

The roles of ILF3 in LPS-stimulated macrophages were next studied by knockdown experiments. As anticipated, transfection of sh-ILF3 resulted in a remarkable reduction of ILF3 in RAW264.7 cells (Fig. 4a). LPS-upregulated ILF3 was attenuated by sh-ILF3 at both mRNA and protein levels (Fig. 4b, c). LPS-induced changes of M1/M2 ratio were reversed by sh-ILF3 as detected by flow cytometry (Fig. 4d). The results of qRT-PCR and western blot further supported these findings in which LPS-mediated changes of M1/M2 markers were reversed by sh-ILF3 (Fig. 4e, f). Taken together, these findings indicate that silencing of ILF3 promotes M2 polarization in LPS-stimulated macrophages.

### ILF3 is required for the stabilization of NEAT1 through direct interaction

**Fig. 5:**
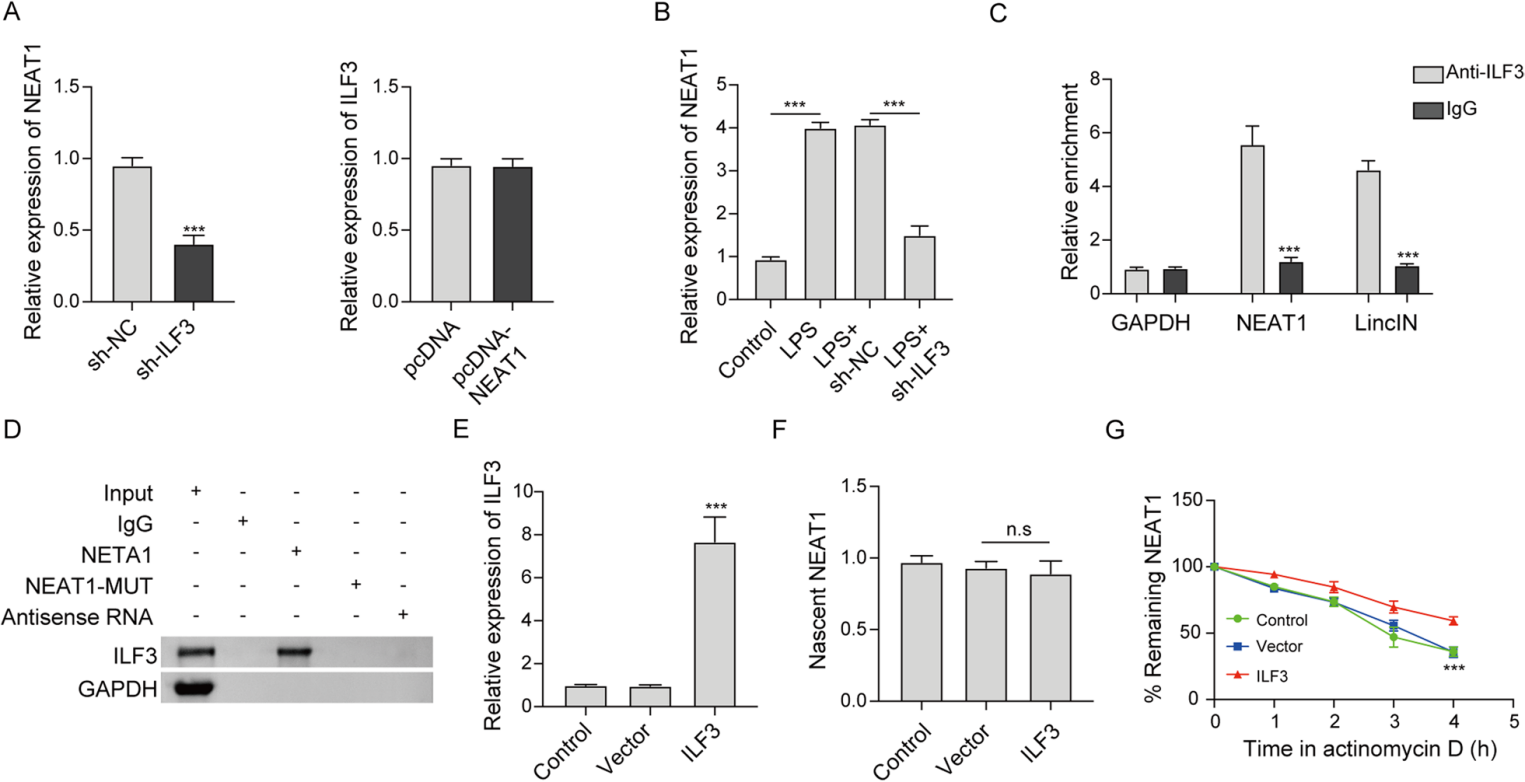
ILF3 is required for the stabilization of NEAT1 through direct interaction. **a**, **b** The levels of NEAT1 and ILF3 in RAW264.7 cells were detected by qRT-PCR. The interaction between ILF3 and NEAT1 in RAW264.7 cells was assessed by RIP (**c**) and RNA pull-down assays (**d**). Normal IgG served as negative control for RIP assay. Antisense RNA acted as a negative control for RNA pull-down assay. **e** The mRNA level of ILF3 in RAW264.7 cells was detected by qRT-PCR. **f** Nascent NEAT1 was evaluated by nascent RNA capture assay. **g** The stability of NEAT1 in RAW264.7 cells was assessed by RNA stability assay. ***, *P* < 0.001

qRT-PCR revealed that knockdown of ILF3 dramatically reduced NEAT expression in RAW264.7 cells (Fig. 5a). Conversely, NEAT1 overexpression had no effect on ILF3 expression (Fig. 5a), suggesting that ILF3 functions as an upstream molecule which positively regulates NEAT1 expression in macrophages. As expected, silencing of ILF3 counteracted LPS-mediated upregulation of NEAT1 (Fig. 5b). RIP assay showed that antibody against ILF3 markedly immunoprecipitated NEAT1 (Fig. 5c). The direct association between ILF3 and NEAT1 was further confirmed by RNA pull-down assay in which the biotinylated NEAT1 successfully pulled down ILF3, while this interaction was abrogated by mutated NEAT1 (Fig. 5d). Overexpression of ILF3 led to a remarkable induction of ILF3, whereas it exerted no effect on nascent NEAT1 (Fig. 5e, f). RNA stability assay revealed that ILF3 overexpression greatly enhanced the stability of NEAT1 in the presence of actinomycin D (Fig. 5g), suggesting that ILF3 extends the half-life of NEAT1 in macrophages. These data indicate that ILF3 plays an indispensable role in stabilizing NEAT1.

### ILF3 impairs M2 polarization of RAW264.7 cell via modulating NEAT1

**Fig. 6:**
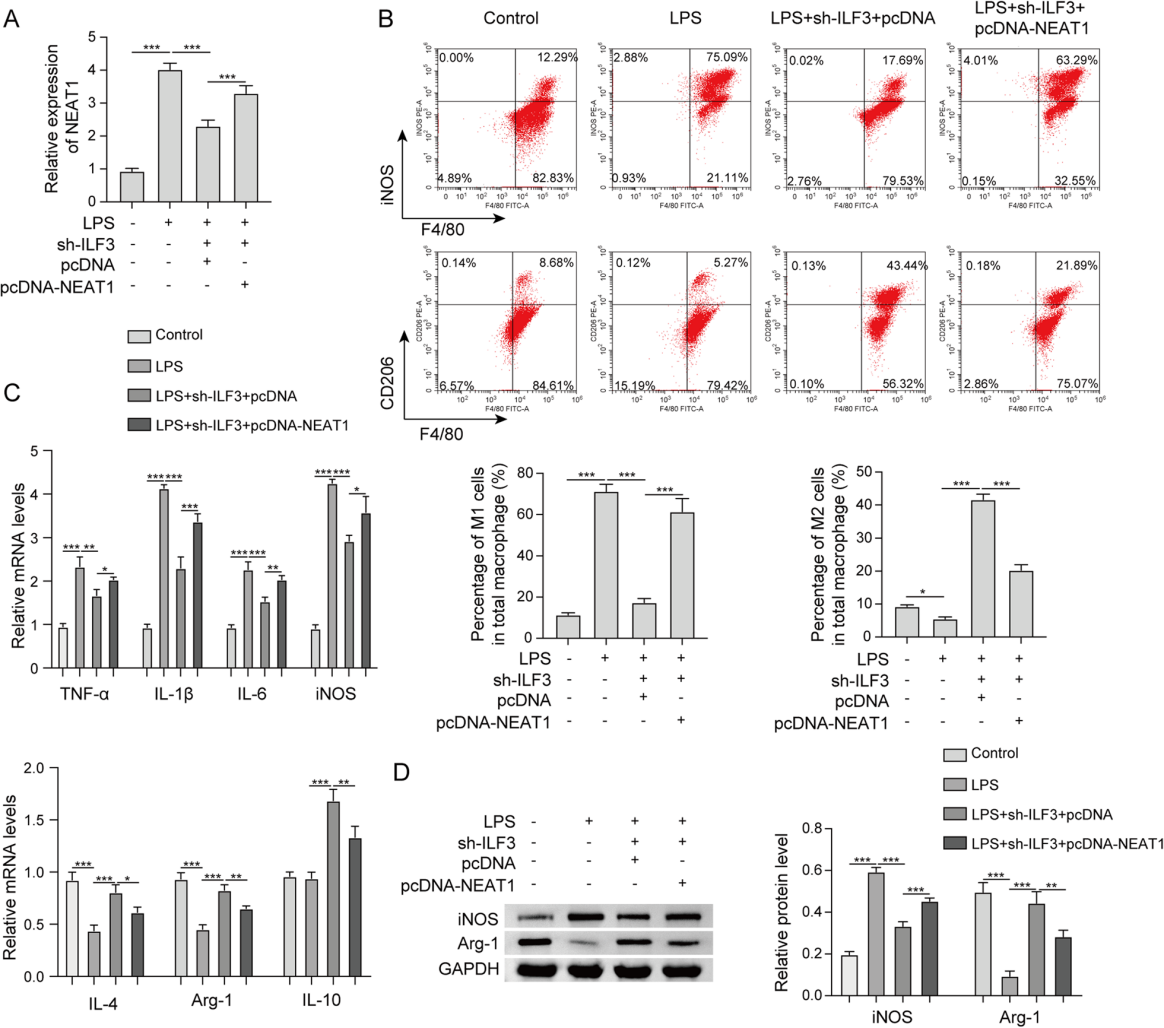
ILF3 impairs M2 polarization of RAW264.7 cell via modulating NEAT1. **a** The level of NEAT1 in RAW264.7 cells was detected by qRT-PCR. **b** The percentages of M1/M2 macrophages were analyzed by flow cytometry. **c** The mRNA levels of M1 and M2 markers in RAW264.7 cells were detected by qRT-PCR. **d** The protein levels of iNOS and Arg-1 in RAW264.7 cells were detected by western blot. *, *P* < 0.05, **, *P* < 0.01, ***, *P* < 0.001

To evaluate the function of ILF3/NEAT1 axis, functional experiments were next conducted in LPS-stimulated macrophages. LPS-induced NEAT1 was rescued by sh-ILF3, and this rescue effect was further abrogated by NEAT1 overexpression (Fig. 6a). Flow cytometry revealed that LPS-induced changes of M1/M2 ratio was reversed by ILF3 knockdown, while the effect of sh-ILF3 was remarkably attenuated in LPS + sh-ILF3 + pcDNA-NEAT1 group (Fig. 6b). Consistently, qRT-PCR and western blot showed that lack of ILF3 counteracted LPS-induced changes of M1/M2 markers, whereas these effects were partially reversed by NEAT1 overexpression in RAW264.7 cells (Fig. 6c, d). Functional studies indicate that ILF3 impairs M2 polarization of RAW264.7 cell via modulating NEAT1.

### ATF3 is a transcriptional repressor of ILF3.

**Fig. 7:**
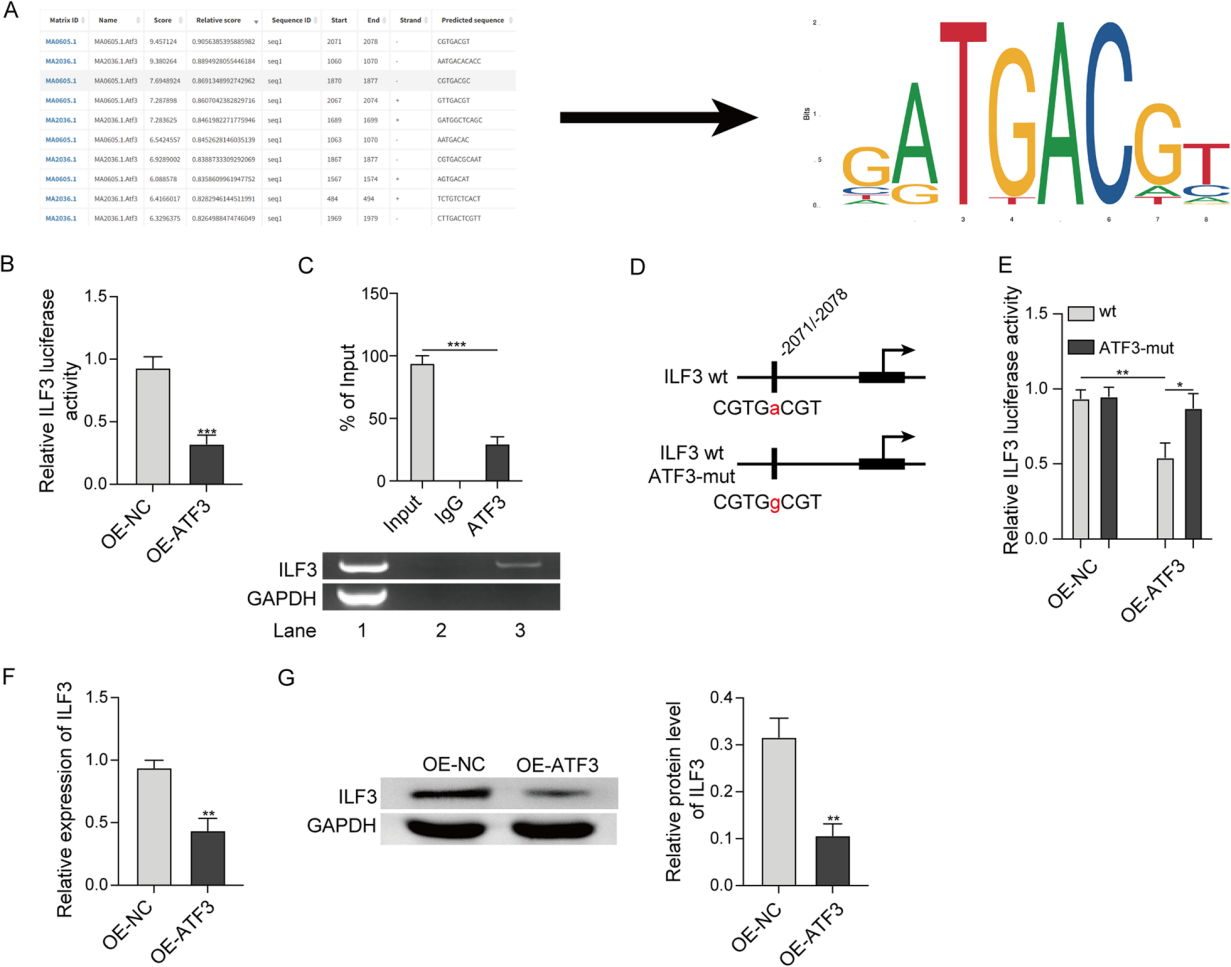
ATF3 is a transcriptional repressor of ILF3. **a** The potential ATF3 binding sites at ILF3 promoter were predicted by JASPAR database. **b**, **d**, **e** The relative luciferase activity was measured by luciferase assay. **c** The direct interaction between ATF3 and ILF3 promoter was assessed by ChIP assay. The mRNA (**f**) and protein (**g**) level of ILF3 were detected by qRT-PCR and western blot, respectively. *, *P* < 0.05, **, *P* < 0.01, ***, *P* < 0.001

JASPAR database predicted the potential ATF3 binding sites at ILF3 promoter (Fig. 7a). The luciferase construct containing the wild-type or mutated p(-2071/-2078) of ILF3 promoter was generated. Co-transfection of OE-ATF3 and ILF3 WT resulted in a dramatic reduction of luciferase activity, while ILF3 MUT did not show obvious change (Fig. 7b, d, e). In line with this finding, ChIP assay revealed that anti-ATF3 antibody successfully enriched ILF3 promoter (Fig. 7c), suggesting the direct interaction between ATF3 and ILF3 promoter. Additionally, overexpression of ATF3 decreased ILF3 expression in RAW264.7 cells at both mRNA and protein levels (Fig. 7f, g). These findings indicate that ATF3 suppresses ILF3 expression at transcriptional level.

### ATF3 exerts its biological functions via ILF3/NEAT1 axis

**Fig. 8:**
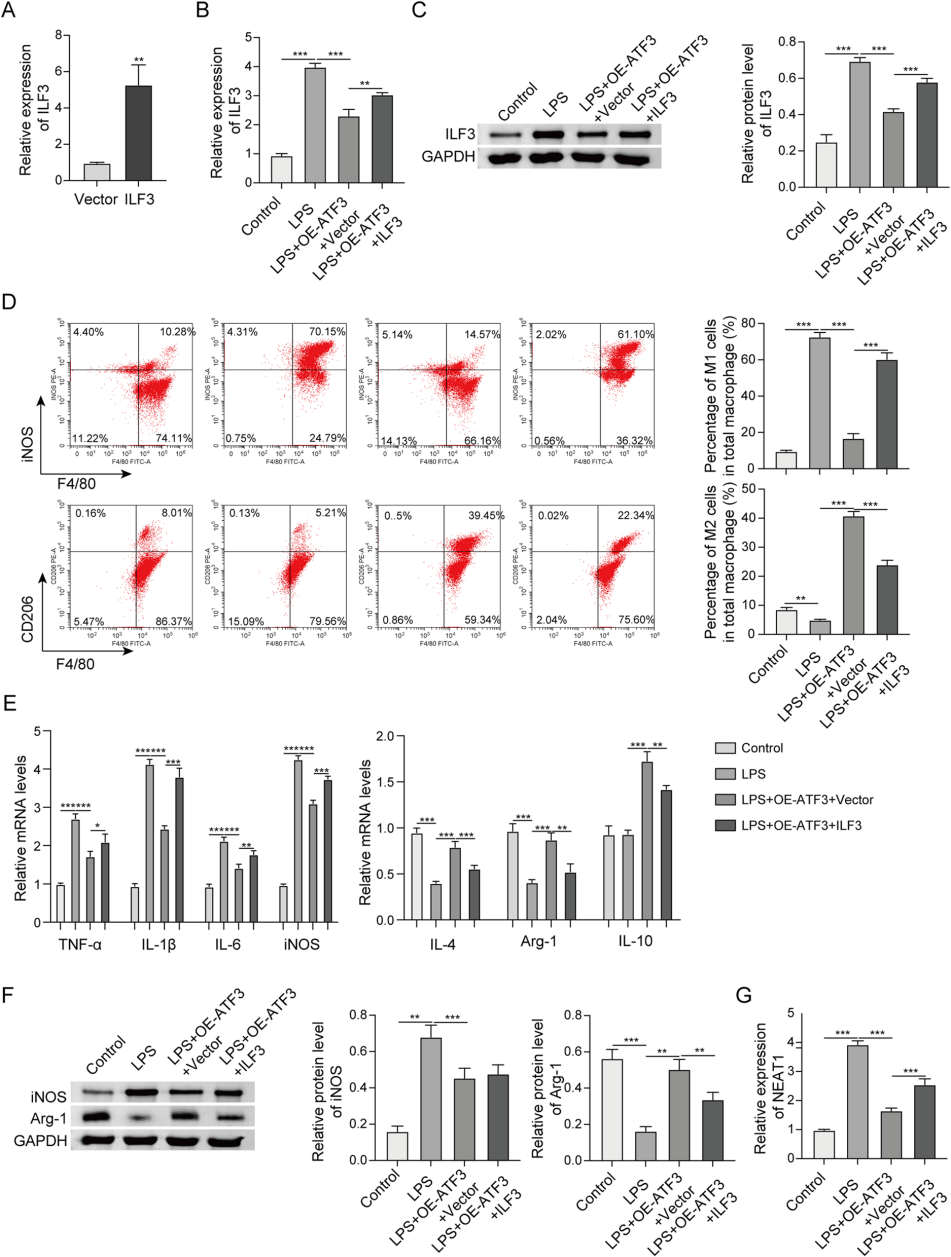
ATF3 exerts its biological functions via ILF3/NEAT1 axis. **a**, **b** The level of ILF3 in RAW264.7 cells was detected by qRT-PCR. **c** The protein level of ILF3 in RAW264.7 was detected by western blot. **d** The percentages of M1/M2 macrophages were analyzed by flow cytometry. **e** The mRNA levels of M1 and M2 markers in RAW264.7 were detected by qRT-PCR. **f** The protein levels of iNOS and Arg-1 in RAW264.7 were detected by western blot. **g** The level of NEAT1 in RAW264.7 cells was detected by qRT-PCR. *, *P* < 0.05, **, *P* < 0.01, ***, *P* < 0.001

We next sought to delineate the biological roles of ATF3/ILF3/NEAT1 axis in LPS-stimulated macrophages. qRT-PCR showed that transfection of ILF3 overexpression construct caused a marked increase of ILF3 in RAW264.7 cells (Fig. 8a). LPS-increased ILF3 expression was attenuated by ATF3 overexpression, and a rebound of ILF3 was observed in LPS + OE-ATF3 + ILF3 group (Fig. 8b, c). Moreover, LPS-induced changes of M1/M2 ratio were reversed by ATF3 overexpression, and co-transfection of ATF3 and ILF3 overexpression plasmids abrogated this effect as detected by flow cytometry (Fig. 8d). Furthermore, LPS-induced changes of M1/M2 markers were counteracted by ATF3 overexpression, and the effects of ATF3 were further abolished by ILF3 overexpression (Fig. 8e, f), indicating that ILF3 functions as a downstream effector in ATF3-mediated M2 polarization. qRT-PCR further showed that ATF3 overexpression attenuated LPS-mediated NEAT1 upregulation, and ILF3 overexpression led to a rebound of NEAT1 in LPS + OE-ATF3 + ILF3 group (Fig. 8g). Collectively, these findings suggest that ATF3 promotes M2 polarization via ILF3/NEAT1 axis.

### The regulatory network of ATF3/ILF3/NEAT1 in the in vivo model of sepsis

**Fig. 9:**
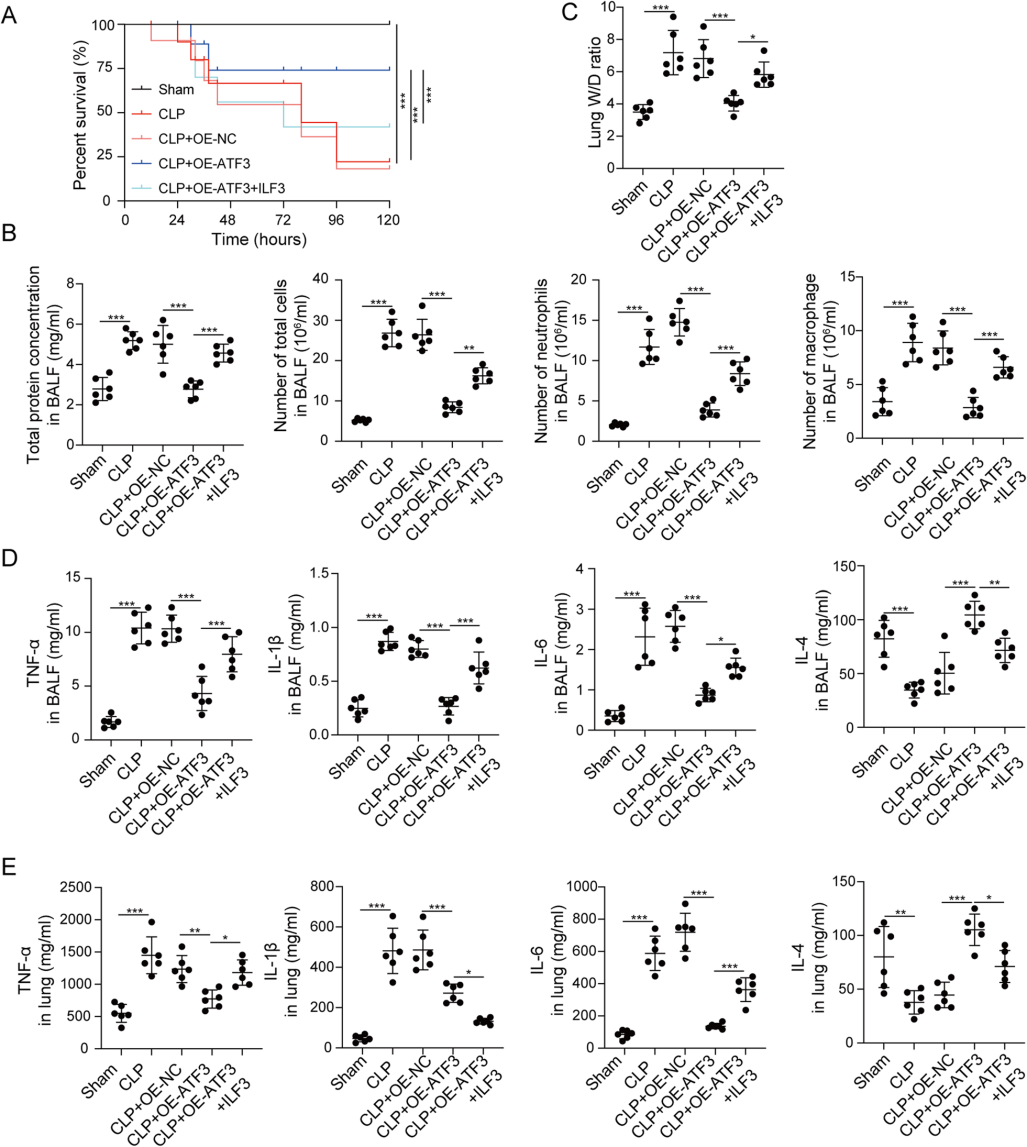

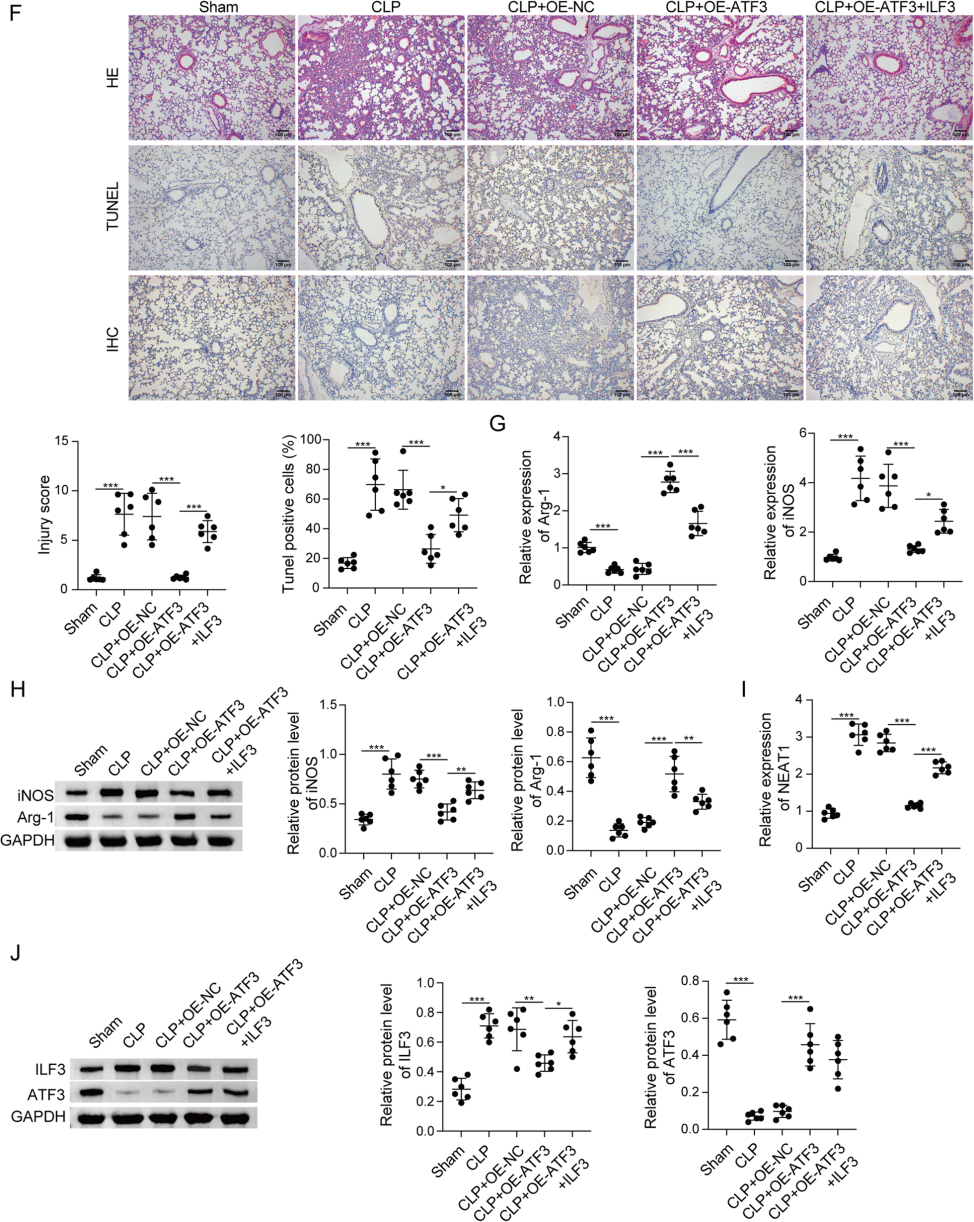
The regulatory network of ATF3/ILF3/NEAT1 in the in vivo model of sepsis. **a** Survival analysis of mice in different groups. **b** Analysis of total protein concentration, numbers of total cells, neutrophils and macrophages in BALF. **c** Lung W/D ratio evaluation. **d**, **e** The levels of cytokines in BALF and lung tissues were measured by ELISA assay. **f** Histological changes of lung tissues were examined by H&E staining. Cell apoptosis in lung tissues was assessed by TUNEL assay. The immunoreactivity of CD163 was detected by IHC. The mRNA (**g**) and protein (**h**) levels of Arg-1 and iNOS in lung tissues were detected by qRT-PCR and western blot, respectively. **i** The level of NEAT1 was detected by qRT-PCR. **j** The protein levels of ATF3 and ILF3 were detected by western blot. *, *P* < 0.05, **, *P* < 0.01, ***, *P* < 0.001

**Fig. 10:**
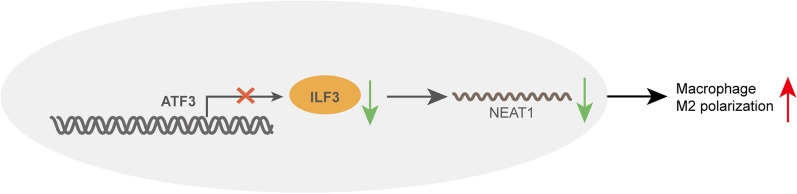
Schematic drawing of ATF3/ILF3/NEAT1 axis in M2 polarization. ATF3 transcriptionally suppresses ILF3 expression, thus impairing the stability of NEAT1, ultimately promoting M2 polarization

The in vitro findings were next validated in CLP-induced sepsis model. Mice were randomly divided into five groups: Sham, CLP, CLP + OE-NC, CLP + OE-ATF3 and CLP + OE-ATF3 + ILF3. All sham mice survived within 168 h, while the survival rates of CLP and CLP + OE-NC mice reduced to be inferior to 25%. ATF3 overexpression exhibited a dramatic protective effect on the survival of CLP mice, whereas no protective effect was found in CLP + OE-ATF3 + ILF3 group (Fig. 9a). CLP remarkably increased total protein concentration, numbers of total cells, neutrophils and macrophages in BALF, and a rescue effect was observed in CLP + OE-ATF3 group. Overexpression of ILF3 partially attenuated this protective effect of ATF3 on total protein concentration and blood counts in CLP mice (Fig. 9b). Analysis of lung W/D ratio revealed that CLP resulted in severe pulmonary edema, and ATF3 overexpression markedly alleviated lung edema. As expected, the protective effect of ATF3 was abolished by ILF3 overexpression (Fig. 9c). ELISA assay showed that LPS greatly increased the levels of TNF-α, IL-1β and IL-6, but decreased IL-4 level in BALF and lung tissues, while the induction of these cytokines were reduced by ATF3 overexpression. Additionally, ILF3 attenuated the protective effects of ATF3 on cytokine secretion (Fig. 9d, e). The histological changes of lung tissues were then examined by H&E staining. As shown in Fig. 9f, compared with sham mice, CLP induced alveolar congestion and the infiltration of inflammatory cells in lung tissues. Consistently, the CLP-induced pathological changes were alleviated by ATF3 overexpression, and this effect was partially abolished by ILF3 overexpression (Fig. 9f). TUNEL assay showed a similar pattern in which the degree of apoptosis in CLP, CLP + OE-NC or CLP + OE-ATF3 + ILF3 group was more severe (Fig. 9f). By contrast, the immunoactivity of macrophage-specific marker CD163 was more prominent in sham and CLP + OE-ATF3 groups (Fig. 9f). Moreover, the M2 marker Arg-1 and the M1 marker iNOS in lung tissues were decreased and increased by CLP, respectively. The CLP-induced changes of these two markers were reversed by ATF3 overexpression, and the effects of ATF3 were further attenuated by ILF3 overexpression (Fig. 9g, h). As anticipated, CLP downregulated ATF3 expression, but upregulated ILF3 and NEAT1 expression in lungs. Overexpression of ATF3 and ILF3 successfully increased their expression in vivo (Fig. 9i, j). ATF3-mediated downregulation of ILF3 was counteracted by ILF3 overexpression, accompanied with a rebound of NEAT1 in CLP + OE-ATF3 + ILF3 group (Fig. 9i). Taken together, these data suggest that ATF3/ILF3/NEAT1 axis is implicated in M2 polarization in CLP-induced sepsis model. ATF3 transcriptionally suppressed ILF3 expression to impair NEAT1 stability, thereby promoting M2 polarization (Fig. 10).

## Discussion

Sepsis is the most common cause of ALI (Matthay et al. 2012). A complicated network of cytokines and chemokines produced by diverse cell types in lungs is implicated in sepsis-induced ALI (Patel et al. 2018). Despite anti-inflammatory drugs and lung-protective ventilation have improved clinical outcomes of ALI, the prognosis of ALI patients remains unfavorite and the mortality rate is still ~ 40% (Memtsoudis et al. 2012). In recent years, researchers have focused on the roles of circular RNAs (circRNAs) in sepsis-induced ALI. For instance, previous study in our lab has illustrated that circEXOC5 exacerbates ferroptosis by modulating PTBP1/ACSL4 axis in sepsis-induced ALI (Wang 2022). Additionally, emerging evidence indicates the pivotal role of macrophage polarization in sepsis (Liu et al. 2014; Murray 2017). In the current study, we reported that ATF3 protected against sepsis-induced ALI by promoting M2 polarization via ILF3/NEAT1 axis, shedding light on the targeted therapeutic strategies for sepsis.

It is well-accepted that M1 macrophages is triggered by LPS and IFN-γ, and characterized by high expression of pro-inflammatory cytokines, such as TNF-α, IL-1β and IL-6. By contrast, M2 macrophages express anti-inflammatory cytokines, such as IL-4 and IL-10 (Murray 2017). Moreover, enzymes responsible for arginine metabolism are differentially expressed in M1/M2 macrophages in which iNOS is elevated in M1 macrophages, and Arg1 is increased in M2 macrophages (Galvan-Pena and O'Neill 2014). In consistent with previous findings, we demonstrated that ATF3 was decreased in PBMCs derived from patients with sepsis and in LPS-stimulated RAW264.7 cells (Luo et al. 2021; Hoetzenecker et al. 2011). In addition, a study has revealed that ATF3 overexpression promotes M2 polarization, accompanied with increased expression of Arg-1, CD163, Mrc-1 and PPARγ, as well as the decreased expression of MCP-1, CD16, iNOS and TNF-α (Sha et al. 2017). In accordance with these findings, our data confirmed that ATF3 overexpression increased the percentage of F4/80^+^CD206^+^ M2 macrophages. It also decreased the expression of iNOS and pro-inflammatory cytokines TNF-α, IL-1β and IL-6, but increased the levels of Arg-1 and anti-inflammatory cytokines IL-4 and IL-10 in vitro and in vivo. Consistently, overexpression of ATF3 remarkably improved survival of CLP mice and alleviated CLP-induced hematological dysfunction and histological damage in vivo. A recent study has demonstrated that AUF1 inhibits ferroptosis to ameliorate sepsis-induced ALI through modulating NRF2 and ATF3 (Wang et al. 2022), suggesting that ATF3 also exerts its protective role in sepsis-induced ALI by regulating ferroptosis. The crosstalk between ATF3-regulated macrophage polarization and ferroptosis merits further investigation.

Accumulating evidence indicates that lncRNAs expressed in macrophages are implicated in the regulation of pro- and anti-inflammatory processes (Chen et al. 2017). NEAT1, located on chromosome 11q13.1, is associated with a variety of inflammatory diseases, including sepsis (Chen 2018; Xia et al. 2018; He et al. 2019; Wang et al. 2019a). More importantly, NEAT1 is upregulated in patients with sepsis, and associated with elevated inflammation, increased sepsis risk and poor survival (He et al. 2019). A number of studies have illustrated that NEAT1 regulates inflammatory response in sepsis, thereby modulating septic complications, such as sepsis-induced myocardial, kidney or lung injury (Wang et al. 2019b; Wang et al. 2020; Feng et al. 2020; Lv et al. 2021). Recently, several independent research groups reported the critical role of NEAT1 in M2 macrophage polarization (Gao et al. 2020; Zhang et al. 2020; Liu et al. 2021). For instance, NEAT1/miR-224-5p/IL-33 network regulates A1 astrocyte activation through modulating M2 polarization (Liu et al. 2021). In line with the previous findings, NEAT1 was elevated in septic patients and LPS-stimulated macrophages, and identified as a downstream effector involved in ATF3/ILF3-mediated M2 polarization in this study. We also reported that ILF3 played an indispensable role in maintaining NEAT1 stability. Mechanistic studies have demonstrated that NEAT1 and miRNA work in concert to regulate inflammation in sepsis. For instance, NEAT1 is negatively correlated with miR-124 in sepsis, and NEAT1/miR-124 axis correlates with different clinical parameters of sepsis (He et al. 2019). NEAT1 exacerbates sepsis-induced ARDS via miR-27a/PTEN axis (Lv et al. 2021). The molecular mechanism by which NEAT1 contributes to M2 polarization in sepsis need in-depth investigation in the future study.

ILF3 has been identified as a direct target of miR-215-5p. Interestingly, miR-215-5p protects against sepsis-induced myocardial inflammation in LPS-treated H9c2 cells through targeting ILF3 and LRRFIP1. ILF3 is also found as a mRNA stabilizer of LRRFIP1 (Yao et al. 2020). In addition to the crucial role of ILF3 in mRNA stability, we reported that ILF3 was also required for maintaining lncRNA NEAT1 stability. Gain- and loss-of function experiments unequivocally demonstrated the pivotal role of ILF3 in M2 polarization in vitro and in vivo. We demonstrated for the first time that ATF3 suppressed ILF3 expression transcriptionally, thereby impairing NEAT1 stability in LPS-stimulated macrophages. The ATF3/ILF3/NEAT1 axis ameliorated sepsis-induced ALI via modulating M2 polarization.

## Conclusion

In summary, we reported that ATF3 triggered M2 macrophage polarization to protect against sepsis-induced lung injury through ILF3/NEAT1 axis. Our findings might unravel a specific ATF3/ILF3/NEAT1 mechanism and provide promising therapeutic targets and profound implications for targeted therapy of sepsis.

## Data Availability

The datasets used or analyzed during the current study are available from the corresponding author on reasonable request.

## Abbreviations

ALI: Acute lung injury
ARDS: Acute respiratory distress syndrome
Arg-1: Arginase-1
ATCC: American type culture collection
ATF3: Activating transcription factor 3
AUF1: AU-rich element-binding factor 1
BALF: Bronchoalveolar lavage fluid
ChIP: Chromatin immunoprecipitation
circRNAs: Circular RNAs
CLP: Cecal ligation and puncture
CREB: ATF/cAMP response element-binding
H&E: Hematoxylin & eosin
IFN-γ: Interferon-γ
IHC: Immunohistochemistry
IL-1β: Interleukin-1β
ILF3: Interleukin enhancer binding factor 3
iNOS: Inducible nitric oxide synthase
lncRNA: Long non-coding RNA
LPS: Lipopolysaccharide
Mrc-1: Mannose receptor C type 1
NEAT1: Nuclear enriched abundant transcript 1
NRF2: Nuclear factor E2-related factor 2
PBMC: Peripheral blood mononuclear cell
PPI: Protein-protein interaction
PPARγ: Peroxisome proliferator-activated receptor γ
RIP: RNA immunoprecipitation
ROS: Reactive oxygen species
TNC: Tenascin-C
TNF-α: Tumor necrosis factor-α
